# Tuberculosis

**Published:** 1909-12

**Authors:** Robert Powell

**Affiliations:** Denver, Colorado


### Tuberculosis.

Doctor Hull's paper in the September number of the "Red Back" set me to thinking, and the vast importance of the subject impels me to do a little writing. So many consumptives have regained health in the broad, sunlit plains of Texas, and the difficulties in the way of enforcing a law of exclusion are so insuperable, that the medical profession of that State may expect to be called on to care for many of the weak lungs developed in the crowded cities of the North. The therapeutic deductions to be made from some points in Dr. Hull's valuable paper may be, therefore, worth considering.

The most significant sentence in this paper is that where the author tells us that tubercular infection does not result necessarily from the presence of a few bacilli, but that only under "certain conditions" do they set up the tubercular process. It seems advisable that we should study carefully these conditions. He says, "Infection requires a sufficient quantity of virulent bacilli and a depressed condition of the tissues."

If only a few bacilli are taken into the body the natural forces of the organism can dispose of them and no tubercular focus is established. These natural forces must be sought in the blood and lymph circulation which penetrate to every living cell of the body, for in no other way can we conceive of the protection being exerted wherever it may be needed. Further along Dr. Hull refers in this connection to the leucocytes, so that we presume that he accepts at least as a working hypothesis Metschnikoff's explanation of the phagocytic powers of these bodies. In the development of the line of serum research, with its opsonins, etc., it is to be feared that Professor Vaughan's observations on the power of nuclein to increase the number and activity of these leucocytes have been forgotten. The progress of medical theory is so rapid that before one idea has been digested it is forgotten for a newer suggestion. The application of opsonins has not yet been rendered easy for the rank and file of our profession, but anybody can give nuclein who has a hypodermic syringe.

The second point of non-immunity is the depression of the tissue cells that renders them more vulnerable to the attacks of the bacilli. Here again we look to the blood, which carries to these cells with perhaps an insufficient supply of nutritive material a tinge of depressant toxins derived from retained fecal materials in the large bowel. This again we owe to Metschnikoff, a man who seems to have the faculty of seeing clearly and uttering boldly the most revolutionary sentiments. If, as we know to be the truth, the circulation of such poisonous materials gives rise to skin diseases, headaches, mental and physical depression and many other ailments, it is not unreasonable to assume that it may also render the blood less active against the tubercle bacilli and the cells also less resistant.

At any rate it seems to be good practice to keep the bowels clear and to prevent any possibly infected food lying longer than the proper time anywhere along the alimentary canal. This is, as I say, good practice in all cases, and the improvement that follows even in well marked tubercular cases after the bowels have emptied and disinfected is strongly confirmatory of the theory presented. The longer such infected material remains in the bowel, the more likely it is to develop virulence and infect the body. We know that even when one has partaken of trichinatous pork, if an emetic and brisk cathartic are given promptly the severity of the subsequent symptoms is markedly lessened, or the disease entirely prevented.

In attendance here on various post graduate courses I find that another expedient is being discussed seriously. Many years ago a Frenchman whose name I have forgotten suggested the treatment of pulmonary consumption by injecting carbonic acid and sulphuretted hydrogen gases into the bowels. The method seems to have been forgotten, but there is talk of combating the tubercle bacilli directly by saturating the system with the sulphides of arsenic and lime. It is claimed by the enthusiasts that no "bug" of any description can live in the body of a person from whose skin the fumes of these sulphides are being given off. Further, that no insect will attack such a person, and that by this means we may render ourselves immune during malarial seasons, even though obliged to traverse the lowlands during the night when the malaria is most active. No baneful effects have as yet been noticed from this treatment, which seems well worthy of a practical trial, since if confirmed the benefits it may afford are beyond computation.

ROBERT POWELL, M. D.

Denver, Colorado.

